# A novel and effective approach to generate germline-like monoclonal antibodies by integration of phage and mammalian cell display platforms

**DOI:** 10.1038/s41401-021-00707-3

**Published:** 2021-07-07

**Authors:** Yu-jia Jin, Diao Yu, Xiao-long Tian, Hui-xian Li, Xiao-chao Zhou, Yu Kong, Wei Zhang, Lu Zhang, Cheng Lei, Zhen-lin Yang, Chao Tu, Yan-ling Wu, Tian-lei Ying

**Affiliations:** 1 MOE/NHC Key Laboratory of Medical Molecular Virology, Shanghai Institute of Infectious Disease and Biosecurity, School of Basic Medical Sciences, Shanghai Medical College, Fudan University, Shanghai, 200032 China; 2 Biomissile Corporation, Shanghai, 201203 China; 3 Department of Pulmonary Medicine, Zhongshan Hospital, Fudan University, Shanghai, 200032 China

**Keywords:** phage display, mammalian cell display, germline-like, monoclonal antibody, TIM-3

## Abstract

Phage display technology allows for rapid selection of antibodies from the large repertoire of human antibody fragments displayed on phages. However, antibody fragments should be converted to IgG for biological characterizations and affinity of antibodies obtained from phage display library is frequently not sufficient for efficient use in clinical settings. Here, we describe a new approach that combines phage and mammalian cell display, enabling simultaneous affinity screening of full-length IgG antibodies. Using this strategy, we successfully obtained a novel germline-like anti-TIM-3 monoclonal antibody named m101, which was revealed to be a potent anti-TIM-3 therapeutic monoclonal antibody via in vitro and in vivo experiments, indicating its effectiveness and power. Thus, this platform can help develop new monoclonal antibody therapeutics with high affinity and low immunogenicity.

## Introduction

Over the past decades, monoclonal antibodies (mAbs) have become the most important class of therapeutic biologicals on drug market [1, 2]. The development of mAbs remains a key issue in meeting the world’s profound need for biological drugs [3]. Among therapeutic antibodies, antibody affinity plays an important role in biological efficacy [4], and higher antibody affinity typically allows for lower dosage. Generating high-affinity antibodies against important drug targets for clinical use remains a challenging task.

Development of therapeutic antibodies has been accelerated by in vitro antibody selection technologies, which permits rapid generation of millions of clones [5–8]. Phage display is the first and the most widely used platform for the discovery of fully human antibodies [9, 10]. It allows for affinity enrichment selections from an extremely large collection of antibodies with sequences of up to 10^11^ in size [11–14]. One inherent limitation of phage display, however, is that panning a non-immune phage display antibody library sometimes results in the selection of antibodies with *K*_D_ ranges >100 nM [15, 16], so it is frequently not sufficient for effective clinical use [16, 17]. Furthermore, antibodies expressed on phages do not undergo normal mammalian posttranslational modifications. This drawback renders a step of conversion to whole IgG molecules and the expression of the candidate clones in mammalian cells necessary for further characterization of their biological activities. Most importantly, the panning process of phage display selection is a black box process, which cannot guarantee the quality of the output clones during the process [18–20]. A possibility to address one of these issues is to use mammalian cell display, which possesses intrinsic abilities to fold and glycosylate full-length IgG [21]. It enables the display of whole IgG and selection of positive clones with high affinity and other specific biological functions by fluorescence-activated cell sorting (FACS) [7, 8, 22–24], allowing for highly controlled and real-time selection of IgG. These properties render mammalian cell display extremely attractive and potent for antibody therapeutics development. However, one major limitation of this method is the reduced library diversity it allows compared to phage display [8]. The lower transformation efficiency of mammalian cells has been considered a significant barrier to the construction of highly diverse libraries.

As described above, both phage and mammalian cell display have inherent limitations that preclude the successful selection of antibodies with desired affinities and low immunogenicity. To efficiently screen for fully functional IgG antibodies against specific targets, we sought to integrate the phage and mammalian cell platforms by combining a pre-enriched antibody output and a FACS step for selection. In general, conventional mammalian cell libraries are generated by directly inserting DNA of antibodies into a mammalian cell vector. On the contrary, the workflow of our new platform starts with a screening and preselection step in a phage library. First, two rounds of phage library panning are performed, and the pre-enriched polyclonal antibody plasmid DNA from the last selection output is purified and cloned into mammalian cell display vectors. Then, specific antibodies are selected through two rounds of FACS sorting.

To validate this new technology, we set out to generate antibodies with high binding affinity for T-cell immunoglobulin and mucin domain-containing protein 3 (TIM-3), which is a promising candidate for cancer immunotherapy [25, 26] that was first reported in 2002 [27]. In this study, by using our newly developed platform, we obtained a novel anti-TIM-3 germline-like mAb named m101, which was found to possess high binding affinity, specificity, and potency in in vitro and in vivo experiments. We show that this novel approach, which integrates phage and mammalian cell display platforms, can overcome the limitations of said platforms and allow effective and reliable selection of full-length IgG antibodies with germline-like sequences and high binding affinity.

## Materials and methods

### Mice

Six- to eight-week-old female TIM-3 humanized C57BL/6N mice were obtained from the Nanjing Galaxy Biopharma Company, China. Six- to eight-week-old male NSG mice were obtained from Shanghai Model Organisms Center, China. The mice were housed under specific pathogen-free conditions in the Animal Care Facility of Fudan University. All the animal studies were carried out in accordance with institutional guidelines.

### Cell line

TIM-3-overexpressing FCHO cells were generated in our laboratory. Human embryonic kidney Expi 293F cells were purchased from Thermo Fisher. Murine colon cancer cell line MC38, Chinese hamster ovary cell line, and human lymphoma Raji cells were purchased from the Type Culture Collection of the Chinese Academy of Sciences.

### Cell culture

Human lymphoma Raji cells were maintained in RPMI-1640 culture medium (Hyclone) with 10% fetal calf serum (Gibco), 100-units/mL penicillin (Gibco), and 100-μg/mL streptomycin (Gibco), in a 37 °C incubator with 5% CO_2_. Murine colon cancer cell line MC38 was maintained in DMEM culture medium (Hyclone) with 10% fetal calf serum (Gibco), 100-units/mL penicillin (Gibco), and 100-μg/mL streptomycin (Gibco), in a 37 °C incubator with 5% CO_2_. TIM-3 overexpressing FCHO cells were maintained in F12 culture medium (Hyclone) with 10% fetal calf serum (Gibco), 100-units/mL penicillin (Gibco), and 100-μg/mL streptomycin (Gibco), in a 37 °C incubator with 5% CO_2_. The cells were expanded in T-175 flasks, harvested by trypsinization on the day of inoculation. The cell viability was tested with Trypan Blue staining and was >95% during the incubation.

### Protein and antibody expression

Overlapping PCR was used to construct a cDNA encoding a fusion protein with TIM-3 extracellular domain and human Ig Fc fragment. The overlapping PCR product was cloned into a PTT expression vector. Plasmids for antibody expression were from *E. coli* and were used for transfection and protein expression. Expi 293F cells were transfected, and the proteins secreted in the medium were purified by Protein A affinity chromatography (GE Healthcare). The homogeneity and purity of the protein preparations were verified by SDS-PAGE. Protein concentration was measured by reading 280-nm absorbance.

Both the H and L chain genes of anti-TIM-3 antibodies were cloned into PTT expression vectors. The H and the L chain vectors were co-transfected into 293F cells using the Expi293 expression system (Thermo Fisher Scientific) following the manufacturer’s instructions. Then, IgGs were purified by Protein A affinity chromatography (GE Healthcare). Proteins were dialyzed against phosphate buffered saline (PBS). Protein concentration was here measured with a microplate reader (Biotek).

### Screening of anti-TIM-3-Fc mAb from phage display library

First, we used a large phage display naive human Fab library constructed by using mixed PBMC cDNAs from 40 healthy volunteers to achieve a titer of 1.5 × 10^11^ [28]. TIM-3-Fc-biotin and streptavidin (SA) beads were used for two rounds of magnetic selection. Phages from the library were pre-blocked in 3% milk powder (*w*/*v*) in PBS (MPBS) and incubated with the TIM-3-Fc-biotin in 1% MPBS for 30 min. Next, SA magnetic beads were added and incubated for 1.5 h. The tubes were washed with PBS containing 0.05% Tween 20 (PBST). TIM-3-Fc binding phages were used to infect mid-log phase TG1 *E. coli* at 37 °C for 1 h. Then, TG1 bacteria were grown in 2YT medium containing 100-mg/mL ampicillin and 2% (w/v) glucose at 37 °C. After 2 h, the cells were infected with M13KO7 helper phages (Invitrogen) for 45 min at room temperature. The infected cells were harvested and resuspended into 2YT medium supplemented with 100-mg/mL ampicillin and 100-mg/mL kanamycin, then incubated overnight at 30 °C. The phages were precipitated from the culture supernatant with PEG8000-NaCl and resuspended in sterile PBS for subsequent panning. The enrichment for antigen-specific phages after each round of panning was assessed by polyclonal phage enzyme-linked immunosorbent assay (ELISA).

### Screening of anti-TIM-3-Fc mAb from mammalian cell display library

The genes encoding the variable heavy chain (VH) and variable light chain (VL) of these polyclonal phages were amplified by PCR and purified. Then, the DNA fragments of polyclonal phages were used to construct two mammalian cell display antibody (IgG1) libraries in the FVTM vectors by chain replacement [7]. A light chain replacement library was constructed using VH genes of polyclonal phages and VL genes of PBMCs, and a heavy chain replacement library was constructed using VL genes of polyclonal phages and VH genes of PBMCs. The FVTM vector contains a trans-membrane domain allowing the display of antibodies on the cell surface. The antibody heavy and light chain coding sequences were cloned into FVTM and the resulting vectors were transiently transfected into FCHO cells. The size of the IgG libraries was ~1 × 10^6^. We used a Flp-In system to integrate a single-copy vector into the genome of FCHO cell.

The TIM-3 antigen was labeled with fluorescein isothiocyanate (FITC) and the antibody library cells were labeled with phycoerythrin (PE)-mouse antihuman κ light chain antibody. Then, the cells and TIM-3 proteins were mixed and incubated on the ice for 30 min. The double-positive cells were single-cell sorted by flow cytometry and seeded in 96-well plates. After the cells were overgrown, they were collected, incubated with fluorescently labeled TIM-3 antigen, and checked for their binding affinity to TIM-3 by flow cytometry. The positive cell clones were harvested and resuspended in cell lysis solution (pH 8.0, 10-mM Tris-HCl, 0.1-M EDTA, 0.5% (*w*/*v*) SDS) to obtain the genomic DNA. Next, PCR was used to amplify the VH domains and VL domains for sequence analysis.

### Affinity measurement by FACS

Purified mAbs were directly conjugated to PE fluorescein. TIM-3 overexpressing FCHO cells were resuspended in PBS containing 2% fetal bovine serum (FBS) (2% FBS–PBS). The binding specificity of the mAbs was determined by incubating serial dilutions of the AF488-labeled mAbs with 2 × 10^5^ of TIM-3 overexpressing FCHO cells. PE-stained cells were analyzed on FACS BD Fortessa.

### ELISA

The binding capacity of anti-TIM-3 IgGs was measured by ELISA. ELISA was performed as described. Briefly, TIM-3-Fc was diluted to a final concentration of 2 mg/mL with PBS and plated on Corning half-area 96-well plates (Sigma-Aldrich) (50 μL for each well) at 4 °C overnight. The coating solution was removed, and the remaining protein-binding sites were blocked with 3% (*w*/*v*) bovine serum albumin in PBS at 37 °C for 1 h. Gradient concentration of IgG was added in triplicate. After reacting at 37 °C for 1.5 h, the plate was washed seven times with PBS 0.05% Tween20. Fifty microliters of horseradish peroxidase (HRP)-conjugated goat antihuman IgG fab antibody (Invitrogen) was added to each well and incubated at room temperature for 45 min. The plate was then washed three times with PBS 0.05% Tween20. Finally, ABTS substrate (Invitrogen) was added to each well for colorimetric development for 15 min. The absorbance was read at 405 nm with a plate reader.

For polyclonal phage ELISA, 1 × 10^12^ phages from each round of panning were added to culture plates, incubated at 37 °C for 1.5 h. After washing three times, HRP-conjugated anti-M13-polyclonal antibody was added, and the plates were incubated at 37 °C for 45 min. After three washes with PBST, ABTS was added for color development. The absorbance at 405 nm was determined with a plate reader.

### Mixed lymphocyte reaction assay

Dendritic cells (DCs) were generated by culturing monocytes isolated from PBMCs using a monocyte purification kit (Miltenyi Biotec) in vitro for 7 days with 500-units/mL interleukin-4 and 250-units/mL GM-CSF (R&D Systems). CD4^+^ T cells (1 × 10^5^ cells) and allogeneic DCs (1 × 10^4^ cells) were co-cultured with different concentrations of antibodies. After 5 days, interferon gamma (IFN-γ) secretion in culture supernatants was analyzed using ELISA (R&D Biosciences).

### Induction of CD69 expression by m101

Each reaction was set up in complete culture media (RPMI-1640, 10% FBS, penicillin-streptomycin) with 1 × 10^5^ PBMCs, 0.25 -μg/mL of anti-CD3 mAb (Mouse Anti-Human CD3, BD Biosciences), 0.2-μg/mL of anti-CD28 mAb (Mouse Anti-Human CD28, BD Biosciences), and the tested antibody at the desired final concentration. The complete reaction was incubated for 48 h at 37 °C, then cells were collected by centrifugation (400 × *g* for 5 min) and stained to measure cell-surface expression of CD69. Activated PBMC pellets were resuspended in 95 μL of FACS buffer (PBS, 1% BSA, 0.1% NaN_3_) and 5 μL of FITC-labeled CD69 antibody (BioLegend FN50, 100-μg/mL), followed by a 30 min incubation on ice. Labeled cells were washed twice with fresh FACS buffer by pelleting (400 × *g* for 5 min) and resuspension, and final cell pellets were resuspended in FACS buffer at 1 × 10^6^ cells/mL. Staining was assessed by flow cytometry (Thermo Fisher Scientific, Attune NxT) and the results were analyzed using Flowjo and GraphPad Prism V6 software.

### Antibody-induced internalization assay

TIM-3-overexpressing FCHO cells were seeded on six-well chamber slides (1 × 10^5^ cells per well) and cultured for 24 h prior to treatment and subsequent immunostaining. Cells were then incubated with m101, LY3321367, or irrelevant IgG1 coincubated with 100 nM for 1.5 h at 4 °C. After 1.5 h of incubation at 4 °C, the inoculum was removed and replaced with complete growth medium, and further incubated for 4 h at 37 °C. After the incubation period, unbound antibody was washed off with PBS and cells were fixed in 4% paraformaldehyde for 15 min at room temperature. Cells were then permeabilized in PBS plus 0.2% Triton-X-100 for 10 min, and nonspecific labeling was blocked in PBS plus 5% BSA. Antibodies that were bound to the cell surface and antibodies that had been internalized were visualized by incubating cells with Alexa Fluor 647-labeled goat antihuman IgG (Thermo Fisher Scientific). Nuclei were visualized with DAPI. As a control, cells were fixed after the 4 °C incubation (T0). Coverslips were mounted using ProLong Gold Antifade reagent (Thermo Fisher Scientific) for imaging. High-resolution laser confocal image sections were acquired using a Leica TCS SP8 03040108.

### Surface plasmon resonance assay

The binding kinetic of m101 to TIM-3-Fc protein was measured using the Octet RED 96 system (Fortebio) at 37 °C. The biotin-labeled TIM-3-Fc was loaded onto SA biosensors. The antibody m101, at concentration ranging from 50 to 3.125 nM was bound to TIM-3-Fc. The binding between a ligand immobilized on the biosensor tip surface and an analyte in solution produces changes the thickness of the biological layer, which are measured in real time. Then, the data were fitted to a 1:1 binding model using Octet software.

### In vivo studies

To evaluate the antitumor effect of mAb m101 in vivo, a syngeneic model of mouse colon cancer was prepared by inoculating MC38 cells (2 × 10^5^ cells) subcutaneously into the right flank of 6- to 8-week TIM-3 humanized C57BL/6N mice. Antibody with a dose of 10-mg/kg or PBS, as negative control, was administered intraperitoneally from the day of tumor inoculation (d 0), and every 3 days afterwards over a total of six doses. Tumor volume was calculated using the equation 0.5·*L* × *W*^2^, where *L* and *W* refer to the length and width of the tumor, respectively.

A xenograft tumor model was also established to evaluate the antitumor effect of m101. NSG mice were inoculated subcutaneously with 1 × 10^6^ human lymphoma cells Raji on the right flank at d 0. At d 7, NSG mice were injected with 1 × 10^7^ PBMCs intravenously. Then, the mice were treated intraperitoneally with 10 mg/kg m101, 10 mg/kg LY3321367, or PBS twice a week starting on d 8, and over a total of six doses. Tumor volume was monitored twice weekly as described above.

### Statistical analyses

Statistical analyses were performed by using GraphPad Prism V6 software. Data are expressed as means ± SEM from three independent experiments. Two group comparisons were performed by unpaired Student’s *t* test. Multiple group comparisons were performed by one-way ANOVA followed by the Dunnett’s *t* test. The level of statistical significance was set at *P < 0.05, **P < 0.01, and ***P < 0.001.

## Results

### Screening of phage and mammalian cell libraries against TIM-3

**Fig. 1:**
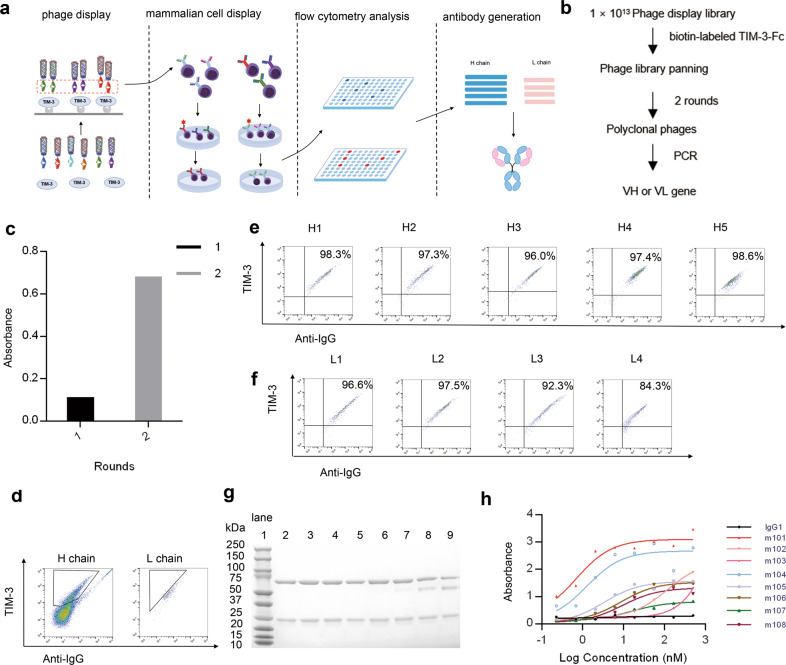
The schematic biopanning process and the characterization of TIM-3-specific mAbs from the antibody library. **a** Flow chart of the anti-TIM-3 mAbs selection strategy combining both phage and mammalian cell antibody display. Two rounds of phage-based panning were performed against TIM-3-Fc protein. Then, the enriched phage genes and genes from PBMC were used to construct mammalian cell display antibody (IgG1) library. Through two rounds of FACS analysis, several positive clones were selected and sequenced. **b** Flow chart of the panning strategy in phage display library. Two rounds of phage-based panning were performed against biotin-labeled TIM-3-Fc protein. The VH and VL genes of polyclonal phages were obtained by PCR. **c** Polyclonal ELISA of phage panning. Approximately 1 × 10^12^ phages from each round of panning were assessed by polyclonal phage ELISA to identify the enrichment of antigen-specific phages. **d**–**f** Illustration of mammalian cell-surface IgG display library selection against TIM-3 by FACS. The TIM-3 antigen was labeled with fluorescein isothiocyanate (FITC) and the antibody library cells were assessed by phycoerythrin (PE)-mouse antihuman κ light chain antibody. **g** The reducing SDS-PAGE analyses of antibodies. The molecular weight markers were used, and antibody molecular weights are indicated to the left of the figures in kDa. Lane 1–9: marker, m101, m102, m103, m104, m105, m106, m107, and m108. **h** Binding of TIM-3-specific mAbs to TIM-3-Fc. TIM-3-Fc was coated on half-area 96-well plates at 4 °C overnight. Gradient concentration of IgG (1 µM to 0.45 nM) was detected. The absorbance was read at 405 nm with a plate reader.

To generate high-affinity mAbs against TIM-3, we developed a novel antibody screening platform that combines phage-based panning and the mammalian cell-based screening (Fig. 1a). We constructed a fully human phage display antibody library from which some mAbs were isolated as previously reported. Here, the first step, consisting of two rounds of phage-based panning (Fig. 1b), was performed against a TIM-3-Fc protein, so a pool of phage clones was produced. The enrichment in TIM-3-Fc-specific phages was confirmed by polyclonal phage ELISA (Fig. 1c), after which those polyclonal phages from the second round of panning were used to infect *E.coli* TG1 and the antibody-expressing plasmids were extracted. Then, a light chain replacement library was constructed using VH genes of polyclonal phages and VL genes of PBMCs, and a heavy chain replacement library was constructed using VL genes of polyclonal phages and VH genes of PBMCs.

To demonstrate the feasibility of high-affinity antibody enrichment by FACS screening, the two cell populations of the antibody display libraries were mixed with non-transfected parental FCHO cells at different ratios, co-stained with 100-ng/mL of specific antigen and anti-kappa chain antibody as indicated, and then analyzed by FACS. Through two rounds of FACS analysis, several positive clones were selected from the libraries (Fig. 1d–f). Analysis by sequence alignment revealed that eight clones had unique sequences. These clones were expressed and purified in 293F cells (Fig. 1g), and finally, a panel of mAbs was identified and characterized based on their binding activity to TIM-3-Fc (Fig. 1h). We further tested the binding specificity of these mAbs to human TIM-3-Fc by ELISA. The results showed that eight mAbs were specific for TIM-3 antigen. Among these, the m101 clone exhibited the best binding ability to TIM-3 antigen. As shown in Fig. 1h, m101 bound to TIM-3-Fc in a dose-dependent manner and with a half maximal effective concentration (EC_50_) of 1.26 nM. Thus, subsequent experiments focused on the characterization and functional analysis of m101.

### Sequence analysis

**Fig. 2:**
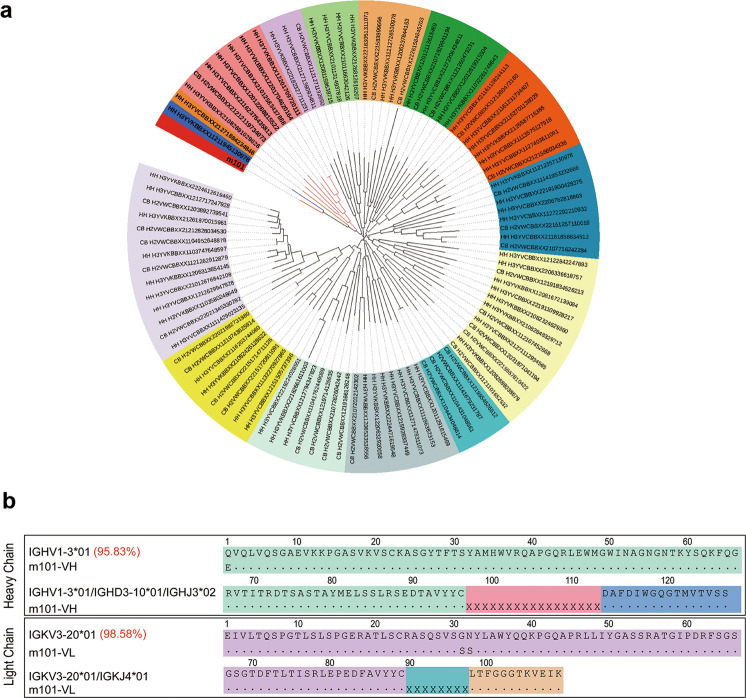
Immunogenetic analysis of m101 mAb. **a** Germline-rooted circular phylogenetic tree of m101-like antibody sequences found in IgM libraries derived from healthy human adults and neonates. The sequences of m101 are shown in red. Phylogenetic tree was constructed using the neighbor-joining method. **b** Immunogenetic analysis of the heavy- and light-chain variable regions of m101 using the IMGT tool. m101 VH gene is derived from the IGHV1-3*01 and m101 VL gene is derived from IGKV3-20*01.

To further characterize the VH sequences of m101, we analyzed in detail the recombination frequency of m101 IGHV with specific IGHD and IGHJ genes in our next-generation sequencing data previously obtained from the naive immunoglobulin M (IgM) repertoires of 33 adult healthy donors [28]. The analysis revealed that among a total of 10,498,301 sequences from healthy adult IgM repertoires, 1063 sequences shared high sequence similarity with m101 (Fig. 2a).

We next performed an immunogenetic analysis of m101 using the IMGT tool to determine to which VH and VL germline genes it was most closely related. This analysis showed that m101 VH gene was derived from the IGHV1-3*01 with which it shared 95.83% identity, while the m101 VL gene was derived from IGKV3-20*01, with which it shared 98.58% identity (Fig. 2b). As shown in Fig. 2b, m101 had limited somatic mutations from its germline predecessors, suggesting it possessed a lower level of immunogenicity [3, 14].

### m101 enhances T-cell activation in vitro

**Fig. 3:**
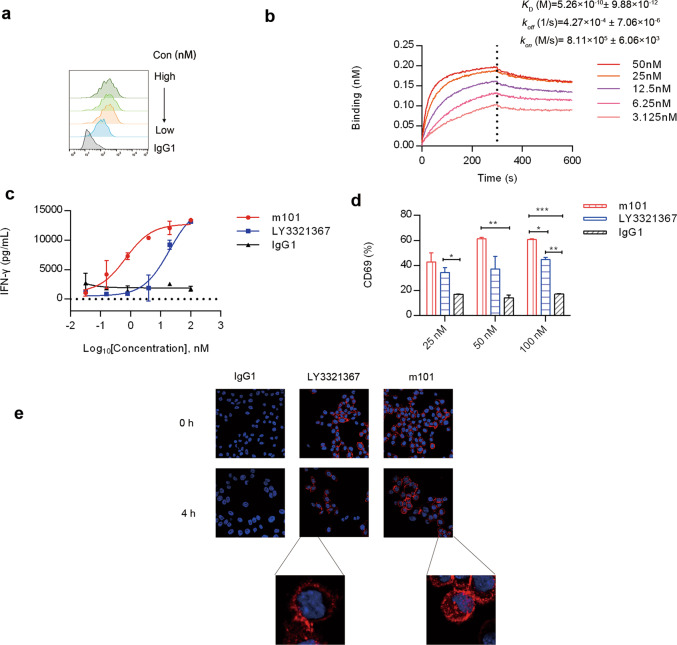
Functional analysis of m101 mAb. **a** m101 binds human TIM-3 expressed on the surface of FCHO cells. The mAb m101 was directly conjugated to PE. The m101 binding specificity was determined by incubating serial dilutions of the AF488-labeled m101 with 2 × 10^5^ of TIM-3 overexpressing FCHO cells. **b** Affinity analysis of m101 mAb to TIM-3 measured by BLI in OctetRED96. The TIM-3-Fc protein was immobilized on activated SA biosensors. The analytes consisted of serial dilution (between 50 and 3.125 nM) of m101 mAb. Binding kinetics was evaluated using a 1:1 Langmuir binding model by ForteBio Data Analysis 7.0 software. **c** Mixed lymphocyte reaction assay to detect IFN-γ secretion. CD4^+^ T cells (1 × 10^5^ cells) and allogeneic DCs (1 × 10^4^ cells) were co-cultured with different concentrations of antibodies. After 5 days, IFN-γ secretion in culture supernatants was analyzed by ELISA. **d** Induction of CD69 expression. PBMCs were activated by anti-CD3 and anti-CD28 antibodies and incubated with the tested antibody at the desired final concentration (25, 50, 100 nM) for 48 h at 37 °C. Then cells were collected and stained by FITC-labeled CD69 antibody. **e** Internalization of anti-TIM-3 mAbs in TIM-3 overexpressing FCHO cells. The cells were permeabilized and stained with AF647 goat antihuman IgG (red) and DAPI (blue).

To further demonstrate the specificity of the m101 in vitro, we performed flow cytometric analysis using 100, 20, 4, or 0.8 nM of m101 directly labeled with AF488 (Fig. 3a). Again, m101 displayed the high binding affinity for TIM-3 expressed by FCHO cells. We further characterized the m101 binding affinity by ForteBio assay. A dose-dependent binding assessment was carried out and relative binding ability values were calculated as described in “Materials and Methods.” Purified m101 was applied to TIM-3-Fc-coated SA sensor (Fig. 3b). According to the results, m101 bound TIM-3-Fc with a dissociation constant (*K*_D_) of 5.26 × 10^−10^ (M). Collectively, these results indicated that mAb m101 possesses good binding affinity to TIM-3 in vitro. To evaluate m101 effect on T-cell activation, we set up a mixed lymphocyte reaction assay using human PBMCs from healthy donors, and PBMC activation was measured using IFN-γ release. M101 alone enhanced IFN-γ production compared to the isotype control (Fig. 3c). A control anti-TIM-3 antibody, and LY3321367 developed by Eli Lilly and Company, also resulted in increase of IFN-γ levels in the cell culture supernatant (Fig. 3c). We further intended to specifically examine T-cell responses in vitro in human PBMCs. Consistent with the results of IFN-γ expression, CD69 expression on PBMCs was also upregulated upon addition of m101 and LY3321367 in a dose-dependent manner (Fig. 3d). Previous reports have shown that anti-TIM-3 antibody can induce an internalization effect of TIM-3 [29], which can shut down the entire TIM-3 mediated signaling regardless of the ligands. To confirm whether m101 was internalized upon incubation at 37 °C, we performed confocal microscopy studies. TIM-3 overexpressing FCHO cells were incubated with LY3321367, m101, or isotype control for 1.5 h at 4 °C, then unbound antibody was washed off with PBS and followed by incubation at 37 °C in complete media to start the internalization process. Finally, cells were subsequently fixed and stained with AF647 antihuman IgG. At time point 0, membrane staining was observed (Fig. 3e). After 4 h of incubation at 37 °C, confocal microscopy confirmed that both m101 and LY3321367 were internalized (Fig. 3e). The presence of intracellular antibodies in the cytoplasm of the cells and a decrease in surface expression were observed (Fig. 3e). These results indicated that our novel screening platform could discriminate antibodies with good biological efficacy.

### Demonstration of the antitumor efficacy of m101 in two humanized mouse models

**Fig. 4:**
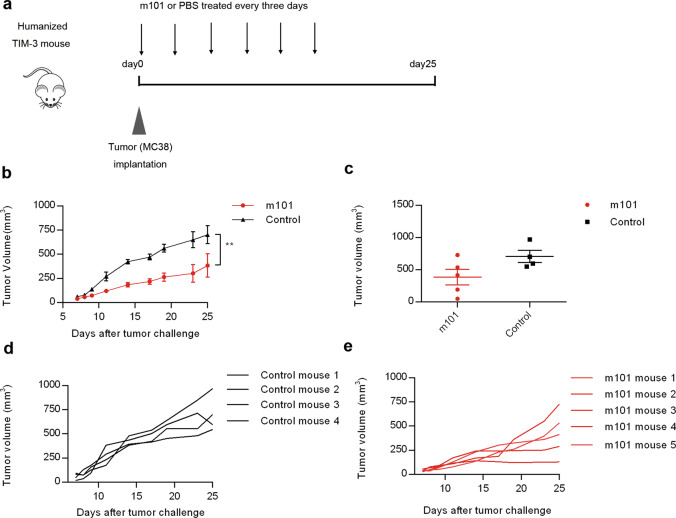
m101 showed potent antitumor activity in TIM-3 humanized mouse bearing MC38 tumors. **a** Experimental setup and treatment schedule are depicted. MC38 cells (2 × 10^5^ cells) were inoculated subcutaneously into the right flank of 6- to 8-week-old TIM-3 humanized C57BL/6N mice. Antibodies or PBS were administered intraperitoneally from the day of tumor inoculation (d 0) and every 3 days afterwards over a total of six doses. **b** Tumor growth in TIM-3 humanized C57BL/6N mice bearing MC38 tumors after treatment with m101 or PBS. M101 or PBS was administered intraperitoneally from the day of tumor inoculation (d 0). Tumor volume was calculated. **c** Tumor volumes at d 25 for individual tumors are depicted. Data are shown as mean values ± SEM. Statistical analysis was performed using Mann–Whitney *U* tests (*P < 0.05; **P < 0.01; ***P < 0.001). Individual tumor growth curves in mice treated with PBS (**d**) or m101 (**e**).

The in vivo antitumor activity of m101 was first tested in humanized TIM-3 mice, subcutaneously implanted with the murine colon cancer MC38 cell line. C57BL/6N TIM-3 humanized mice were inoculated subcutaneously with MC38 cells on d 0 and subsequently treated by intraperitoneal injection of mAb m101 (10-mg/kg) or PBS (Fig. 4a). Tumor growth was assessed from d 7 to d 25. As shown in Fig. 4b, treatment of tumor-bearing mice with m101 (10 mg/kg) induced significant and durable tumor regression compared to the mice of the control group treated with PBS, and individual mouse tumor growth is shown in Fig. 4d, e. However, the results of the last tumor measurement on d 25 showed that there was no significant difference in tumor size between the two groups (Fig. 4c).

**Fig. 5:**
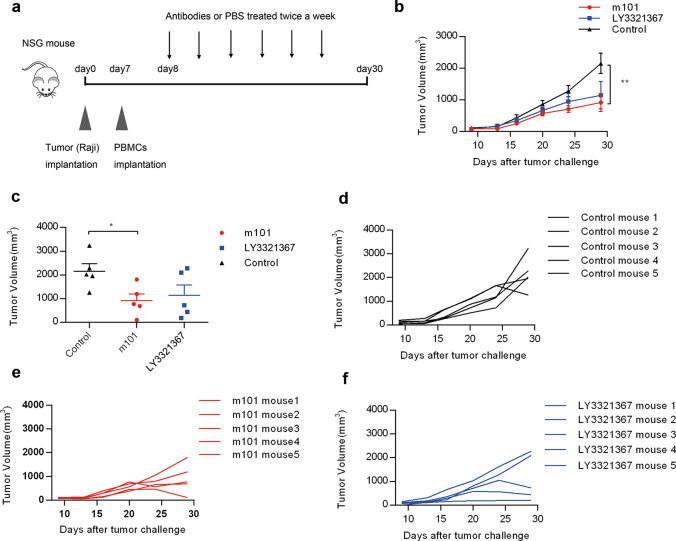
m101 showed potent antitumor activity in NSG mouse bearing Raji tumors. **a** Experimental setup and treatment schedule are depicted. NSG mice were inoculated subcutaneously with 1 × 10^6^ human lymphoma cells Raji on the right flank. 1 × 10^7^ PBMCs were then injected intravenously. The mice were treated intraperitoneally with m101, LY3321367, or PBS twice a week from d 8, for a total of six doses. **b** Tumor growth in NSG mice. Raji tumors were treated with m101, LY3321367, or PBS. Data are shown as mean values ± SEM. Statistical analysis was performed using Mann–Whitney *U* tests (*P < 0.05; **P < 0.01; ***P < 0.001). **c** Tumor volumes at d 29 for individual tumors are depicted. Data are shown as mean values ±SEM . Statistical analysis was performed using Mann–Whitney *U* tests (*P < 0.05; **P < 0.01; ***P < 0.001). Individual tumor growth curves in mice treated with PBS (**d**), m101 (**e**), or LY3321367 (**f**).

To further evaluate the therapeutic activity of m101, we chose a human xenograft NSG mouse model inoculated with PBMCs as an in vivo model (Fig. 5a). The therapeutic efficacy was compared among groups treated with m101, LY3321367, or control (Fig. 5b, c), and the individual mouse tumor growth is shown in Fig. 5d–f. The tumor growth was significantly inhibited in the m101-treated group compared with the control group (Fig. 5b). The results of the last tumor measurement showed a significant difference in tumor size between control and m101-treated mice, while treatment with LY3321367 did not induce significant inhibition of tumor growth (Fig. 5b, c). These results indicated that m101 possesses good antitumor activity in vivo.

## Discussion

The development of therapeutic mAb remains a key issue to fulfill the great needs of biological drugs. Phage display platforms represent powerful in vitro selection techniques for the display and screen of target-specific antibodies. However, it has specific drawbacks, such as inability to display full-length antibody fragment [19, 20] and insufficient affinity for effective clinical use [16]. We sought to resolve this issue by integrating phage and mammalian cell display platforms, and the results suggested that this would overcome the limitations of phage and mammalian cell display platforms and enable effective and reliable selection of full-length IgG antibodies with high binding affinity.

Generating high-affinity antibodies against important drug targets is a critical step in the development of therapeutic mAb because high antibody affinity allows for a lower dosage. In this study, we produced anti-TIM-3 antibody with high affinity by integrating phage and mammalian cell display platforms, using phages to display random libraries and mammalian cells to display full-length IgGs. The advantage of this approach is that it reduced the number of irrelevant clones by two rounds of phage display panning and allowed construction of mammalian cell libraries using phage library outputs, which improved the efficiency. In addition, the selection of mammalian cell display allows highly controlled, real-time selection that can enable a fine discrimination of clones exhibiting different properties such as affinity and features that can be obtained effectively.

To validate this new technology, we chose TIM-3 as the target. It is a promising candidate for cancer immunotherapy first reported in 2002 [27]. TIM-3 is expressed on IFN-γ-producing T cells, FoxP3^+^ Treg cells, and innate immune cells (macrophages and DCs) [27]. The expression of TIM-3 on these cells is required for the maintenance of immunosuppressive environments [30]. Previous data collected in multiple preclinical cancer models indicated that anti-TIM-3 treatment improves T-cell function [31]. Therefore, there is a great significance for the development of antihuman TIM-3 therapeutic antibodies.

Using this strategy, we obtained a fully human m101 antibody with high affinity, binding human TIM-3-Fc with a dissociation constant (*K*_D_) of 5.26 × 10^−10^ (M). All the results of in vitro assay indicated that our novel screening platform could identify antibodies with good biological efficacy. Compared to antibodies, which was induced by high-affinity mutations, germline-like antibodies have a lower level of immunogenicity. Sequence analysis confirmed that m101 is a germline-like antibody with limited mutations, and thus exhibited a lower level of immunogenicity. Taken together, those results can identify germline-like antibodies with high affinity and low immunogenicity using our new platform.

TIM-3 has been reported to have multiple ligands [32–34]. It is becoming increasingly clear that blocking the interactions between TIM-3 and its ligands can enhance antitumor responses [35–37]. It is important that the anti-TIM-3 antibody be able to block these ligand-TIM-3 interactions to prevent an inhibitory signal from TIM-3 [36, 37]. We met this goal by confirmation of the internalization effect of m101, for internalization effect is considered as a completely block effect of TIM-3 signal. Two different in vitro experimental scenarios indicated T lymphocyte activation upon m101 stimulation, and humanized mouse tumor models showed that m101 enhanced antitumor immunity and suppressed tumor growth. Those results showed that m101 can serve as effective cancer therapeutic antibody. Further assessment of m101 is needed to establish the details underlying its mechanism of action.

In conclusion, we created a new full-length antibody screening and selecting strategy. For this strategy, the combination of phage display biopanning step of enrichment for polyclones with binding affinity and two FACS steps of mammalian cell library screening allowed efficient and rapid discovery of IgGs. Using this platform, we identified and characterized a fully human germline-like anti-TIM-3 IgG antibody named m101 with detailed characterization of its in vitro and in vivo efficacy, indicating the complete feasibility of the new strategy. To our knowledge, this is the first report describing a combined phage/mammalian cell display platform, which we believe can be easily applicable for the identification of clinical candidate antibodies.
